# Contagious epididymitis due to *Brucella ovis*: relationship between sexual function, serology and bacterial shedding in semen

**DOI:** 10.1186/s12917-015-0440-7

**Published:** 2015-05-30

**Authors:** Nicole Picard-Hagen, Xavier Berthelot, Jean Luc Champion, Laure Eon, Faouzi Lyazrhi, Maxime Marois, Marceline Peglion, Aude Schuster, Christel Trouche, Bruno Garin-Bastuji

**Affiliations:** Université de Toulouse, INP-ENVT, UMR 1331-Toxalim, F-31076 Toulouse, France; Université de Toulouse, INP-ENVT, UMR 1225-IHAP and UMT Maîtrise de la santé des troupeaux de petits ruminants, F-31076 Toulouse, France; Groupement de Défense Sanitaire des Alpes de Haute Provence, F-04000 Digne les Bains, France; Groupement de Défense Sanitaire des Bouches du Rhône, F-13626 Aix en Provence, France; Université de Toulouse, INP-ENVT, Unité de Biostatistiques, F-31076 Toulouse, France; Fédération Régionale des Groupements de Défense Sanitaire Provence Alpes Côte d’Azur, F-04100 Manosque, France; Paris-Est University/French Agency for Food, Environmental and Occupational Health and Safety (ANSES), EU/OIE/FAO Brucellosis Reference Laboratory, 94701 Maisons-Alfort, France; Present Address: ANSES, European & International Affairs Department, 14 rue Pierre et Marie Curie, F-94701 Maisons-Alfort, Cedex France

**Keywords:** *Brucella ovis*, Semen, Sheep, Genital disease

## Abstract

**Background:**

Contagious Epididymitis (CE) due to *Brucella ovis* (*B. ovis*) is a contagious disease that impairs rams’ fertility due to epididymis, testicle and accessory sexual gland alterations. An increased incidence of CE has been observed in South Eastern France (“PACA” region) since the Rev.1 vaccination against *B. melitensis* has been stopped in 2008. The objective of this study was to evaluate the relationship between the infection by *B. ovis* and the sexual function of rams.

Two-hundred eighteen sexually-mature rams, from 11 seropositive flocks, were submitted to a clinical examination of the genital tract, a semen collection by electro-ejaculation for spermogram and culture, and a serological examination for anti-*B. ovis* antibodies by complement fixation test (CFT) and indirect ELISA (I-ELISA). The relationships between clinical, seminal, bacteriological and serological parameters were studied using the Fisher exact test and a logistic regression model (binomial logit).

**Results:**

*B. ovis* shedding in semen was significantly associated with seropositivity (CFT and I-ELISA; *p* < 0.001 and 0.01 respectively), genital tract alterations (*p* < 0.05) and poor semen quality (*p* < 0.001). Seropositive rams presented significantly more genital tract alterations (*p* < 0.001) and a poor seminal score (*p* < 0.001) than seronegative rams.

**Conclusions:**

Since semen culture is not routinely feasible in field conditions, a control plan of CE should be based, where Rev.1 vaccination is not possible, on both systematic clinical and serological examination of rams, followed by the culling of seropositive and/or genital tract alterations carrier rams.

## Background

Contagious Epididymitis (CE) due to *Brucella ovis* (*B. ovis*) is a contagious disease of worldwide importance that impairs fertility in rams due to epididymis, testicle and accessory sexual gland alterations. More rarely, abortions in ewes and increased perinatal mortality rates may be observed as well [1, 2]. Transmission occurs via passive venereal infection or direct contact [2]. The disease can result in significant economic losses in infected flocks where no control programs are in place, due to reproductive failure, culling of breeding animals as well as ban on trade. In the European Union, no compulsory surveillance of the disease is currently in place in flocks, while neither eradication programme nor compensation scheme for culling animals in infected flocks is foreseen. Nevertheless, in order to avoid the contamination of non-infected areas or flocks through international trade, rams have to undergo serological pre-movement tests [2]. On farms, diagnosis mainly relies on a clinical detection and a serological test when the palpation of testicles reveals lesions or when there is significant infertility in the flock [1, 3].

An increased incidence of CE has been observed in South Eastern France (“PACA” region) since the Rev.1 vaccination against *B. melitensis* has been stopped in 2008 due to eradication of brucellosis in domestic ruminants 5 years before in the whole country [3]. In 2011, the flock prevalence rate (at least 1 seropositive ram per flock) ranged from 5.5 % to more than 50 % according to the area.

The aim of this study was to examine the relationship between *B. ovis* infection, evaluated through clinical, serological and bacteriological findings, and the sexual function, in rams originated from infected flocks of this region where CE appears enzootic.

## Methods

### Animals

Two-hundred eighteen sexually-mature rams from 11 seropositive flocks from the PACA region of South-Eastern France were included in this study in September 2012. Both seropositive and seronegative animals (based on results of tests performed in former spring) were selected by voluntary breeders in a balanced manner, as much as possible. Breeds of rams were as follows: Merinos (*n* = 69), Préalpes du Sud (*n* = 30), meat breeds (Ile de France and Texel; *n* = 91); Merinos cross-breeds (*n* = 15), others (*n* = 13).

### Breeding soundness assessment

For each animal, the following parameters were recorded: age, body condition score (BCS), scrotal circumference (SC, in cm), as well as clinical alterations of the genital tract investigated by palpation and, in suspicious cases, by ultrasonography [4].

The Body Condition Score (BCS) was assessed according to the technique described by Russel [5]. Briefly, the BCS was noted from 0 (extremely emaciated) to 5 (obese) by the palpation of the lumbar region (spinous and transverse processes, muscles and fatty tissues), a BCS of 3 to 4 being considered as optimal during the breeding season [6].

Testicle alterations included asymmetry, indurations, degeneration and/or atrophy, while epididymis alterations included epididymitis, indurations, nodules, hypertrophy and/or cysts.

In order to classify the rams for their breeding potential, a clinical score and a seminal score based upon a point-score system adapted from Ley et al. [7] were calculated for each ram. The clinical score took into account the age, BCS, SC and the presence/absence of clinical signs, as shown in Table 1.

**Table 1:** Clinical score calculation and rams classification

	Note		
	1	4	7
Age (years)	≥5	1	2-4
BCS*	<2	>3	2-3
SC (cm)**	<33	≥33- < 39	≥39
Epididymis/testicle alterations***	Presence (note = 0)	N/A****	Absence (note = 7)
	Clinical score		
	3-12	13-21	22-28
Rams classification	Poor	Fair	Good

*BCS: body condition score (0–5)

**SC: scrotal circumference

***Testicle alterations: asymmetry, indurations, degeneration and/or atrophy; Epididymis alterations: epididymitis, indurations, nodules, hypertrophy and/or cysts

****N/A: not applicable

### Animals’ handling and sample collection

All samples were collected in the frame of the *B. ovis* infection control programme implemented by the Fédération Régionale des Groupements de Défense Sanitaire Provence Alpes Côte d'Azur (FRGDS PACA) as approved by its advisory board and in accordance with the EU (in particular Directive 2010/63/EU) and French regulations regarding ethics and best practices of veterinary care.

Animals’ handling and sample collection were performed by trained technicians of the FRGDS PACA, veterinary surgeons and graduating veterinary students (under the supervision of 2 professors), with the help of the stockbreeders who had brought their own rams and given their informed consent. Blood samples were collected from each ram for serological examinations. Semen was collected by electroejaculation (Electrojac, Ideal® Instruments, MI, USA) for further laboratory examinations. To preserve the welfare of the ram, the stimulation was discontinued if signs of stress or physical discomfort were detected.

### Laboratory examinations

Each blood serum was subjected to both complement fixation test (CFT) and indirect ELISA (I-ELISA; IDEXX *Brucella ovis* Ab Test; Idexx Montpellier, France) as described by Praud et al. [8]. The CF antigen (Anses, Maisons-Alfort, France) was standardised against the International Standard anti-*Brucella ovis* Serum (ISaBoS) and the positivity threshold was 50 international CFT units (ICFTU)/mL according to OIE requirements [2]. I-ELISA was standardized against ISaBoS with the 1/64 pre-dilution of the ISaBoS made up in a negative pool of sera as the minimum detection requirement and the 1/256 dilution at which the standard must be classified as negative. All tests were performed by the same two technicians at the Anses Brucellosis Reference Laboratory (Anses, Maisons-Alfort, France). In a previous work, Praud et al. [8] suggested that depending on the aim of the test performance (screening, diagnosis confirmation, export control) and disease situation (free, enzootic), different cut-offs could be used (30 %, 45 % or 60 %) but found that the 45 % cut-off gave the best concordance with CFT. In this study, I-ELISA results were therefore interpreted either with a unique cut-off of 45 % (negative or positive) or with a double-cut-off of 30 % and 60 % respectively (negative, doubtful or positive).

For the semen examination, the following characteristics were recorded: volume (mL), gross and progressive motility, sperm morphology and abnormalities after eosin-nigrosin staining; sperm concentration (spermatozoa per mL) was measured by spectrophotometry (Accuread, IMV technologies, L'Aigle, France). The seminal score was based on the number of spermatozoa in the ejaculate, the individual motility and the percentage of normal spermatozoa, as shown in Table 2.

**Table 2:** Seminal score calculation and rams classification

	Note		
	1	4	7
Total number of spermatozoa/ejaculate (x10^9^)	= < 1	>1- ≤ 2	>2
Individual motility (%)	= < 30	>30- ≤ 70	>70
Normal spermatozoa (%)	= < 50	>50- ≤ 70	>70
	Seminal score		
	3-9	12-15	18-21
Rams classification	Poor	Fair	Good

Semen cultures for *B. ovis* were performed on samples stored frozen, on non-selective 5 % equine-serum added blood-agar base N°2 (Oxoid, France) in an atmosphere of 5–10 % CO_2_, at the ANSES Brucellosis Reference Laboratory [2].

### Statistical methods

Statistical analysis was performed using the Fisher exact test and a logistic regression model (binomial logit) with the R software (http://www.R-project.org). A threshold of 5 % (*p* < 0.05) was considered as significant.

Kappa statistics were also used to assess the agreement between different diagnostic methods.

## Results

### Clinical and semen laboratory examinations

The clinical and seminal characteristics of the 218 rams are presented in Table 3. In 73 rams, clinical alterations were observed by palpation, mainly on epididymes (*n* = 60; head and tail hypertrophy, indurations and nodules) and testicles (*n* = 13; asymmetry, indurations, degeneration and atrophy); 17 alterations were bilateral, 56 unilateral. 145 rams had no macroscopic alteration of the genital tract. Respectively 26 % and 37 % of rams had a good clinical or seminal score.

**Table 3:** Clinical and seminal characteristics of the 218 rams

Parameter	Mean	SD	Median	Min.	Max.
Age (years)	3.3	1.7	3	1	9
BCS*	2.4	0.7	2.3	1.5	3.8
SC (cm)**	36.4	3.6	34	26	46
Number of spermatozoa/ejaculate (x10^9^)	1.9	1.8	1.3	0.004	8.9
Individual motility (%)	48.9	29.9	57.5	0	90
Normal spermatozoa (%)	70.8	22.5	78.7	2.6	98.5
Clinical score	Seminal score				
	Poor	Fair	Good	Total	
Poor	23	21	12	56	
Fair	31	36	38	105	
Good	12	14	31	57	
Total	66	71	81	218	

*BCS : body condition score; **SC : scrotal circumference

There was a significant relationship between seminal and clinical score (*p* = 0.0084), mainly explained by the presence (or not) of clinical alterations but not by the SC or age.

### Serological results

Detailed serological results are given in Table 4. As expected from the inclusion criteria, about half of animals were effectively seropositive but more clearly in I-ELISA (53.7 %) when a unique cut-off of 45 % was chosen according to Praud et al. [8] than in CFT (37.2 %).

**Table 4:** Distribution of the results (number of rams) and concordance of serological tests

CFT*	I-ELISA (30–60 %)**				
	Negative	Doubtful	Positive	Total	
Negative	95	9	32	136	
Positive	-	1	80	81	
Total	95	10	112	217	
CFT	I-ELISA (45 %)**				
	Negative	Positive	Total		
Negative	100	36	136		Κ = 0.676
Positive	-	81	81		95 % CI [0.584;0.767]
Total	100	117	217		

*One anti-complementary serum not included in the analysis

**Cut-off: 30-60 % or 45 %

Κ: Cohen’s kappa coefficient; 95 % CI = 95 % confidence interval

One serum presented an anti-complementary activity and was removed from the analysis. The two tests gave concordant results for 181 animals (83.0 %) with a higher number of positive results with I-ELISA than with CFT (117 vs. 81). The agreement between CFT and I-ELISA (45 %), evaluated by the Cohen’s kappa coefficient, was good: Κ = 0.676 (95 % CI [0.584; 0.767]).

### Relationship between serological results and clinical alterations

There was a significant relationship between positive serological results and clinical alterations (*p* < 0.001). About 64 % and 81 % of animals with clinical alterations of the genital tract (see above) were positive for CFT and ELISA respectively (Table 5) but only 58 % of animals positive to CFT (47/81) and 50 % of animals positive to I-ELISA (59/117) presented clinical alterations of the genital tract.

**Table 5:** Relationship between serological tests and clinical (testicular and/or epididymal) alterations

Clinical alterations	Serological results					
	CFT*			I-ELISA (45 %)**		
	Negative	Positive		Negative	Positive	
Yes	26	47	*p* < 0.001***	14	59	*p* < 0.001***
No	110	34		87	58	
Total	136	81		101	117	

*One anti-complementary serum not included in the analysis

**Cut-off: 45 %

*** Fisher exact test

### Relationship between serological results and seminal score

Most of I-ELISA-positive animals (83 %) presented a poor or fair seminal score and 40 % of I-ELISA-negative rams presented such a poor or fair seminal score as well (Tables 6 and 7). The Fisher exact test showed a significant relationship between the positive serological status of rams and the low seminal score (*p* < 0.001).

**Table 6:** Pathological changes in spermatozoa

Pathological changes of spermatozoa	Mean ± SD*	Median*	Maximum*
Detached head	10.8 ± 14.6	4.1	80.7
Elongated head	0.1 ± 0.4	0	4.0
Proximal droplet	0.4 ± 1.6	0	19.5
Distal droplet	0.1 ± 0.6	0	6.1
Isolated tail	5.2 ± 7.3	1.6	38.5
Bent tail	7.6 ± 7.2	5.6	51.5
Coiled tail	3.8 ± 4.8	2.0	28.5
Broken tail	1.2 ± 1.5	0.5	8.0

*%

**Table 7:** Relationship between I-ELISA results and seminal score

		Seminal score			
		Poor	Fair	Good	Total
I-ELISA (30-60 %)	Negative	6	32	57	95
	Doubtful		5	6	11
	Positive	60	34	18	112
	Total	66	71	81	218
I-ELISA (45 %)	Negative	6	34	61	101
	Positive	60	37	20	117
	Total	66	71	81	218

### Bacteriological results

Only 198 results were interpretable; for 16 samples, cultures were contaminated by overgrowing microorganisms that made the interpretation impossible; and 4 samples were not tested due to an unsufficient quantity of semen. *B. ovis* was isolated in the semen of 89 rams, *i.e*. 44.95 % of 198 sampled animals with culture results.

There was a significant relationship (*p* < 0.05) between culture results and clinical score (Table 6). Among the parameters determining the clinical score, only BCS (*p* < 0.01) and lesions (*p* < 0.001) influenced significantly the bacteria shedding.

There was a significant relationship (*p* < 0.001) between culture results and seminal score (Table 8).

**Table 8:** Relationship between bacteriology and clinical or seminal score

			Clinical score (*n* = 218)				Seminal score (*n* = 218)			
			Poor	Fair	Good		Poor	Fair	Good	
*B. ovis* culture results (*n* = 198)	Negative	No.	22	54	33	*p* < 0.05**	17	33	59	*p* < 0.01**
		(%)	(10.1)	(24.8)	(15.1)		(7.8)	(15.1)	(27.1)	
	Positive	No.	29	42	18		43	34	12	
		(%)	(13.3)	(19.3)	(8.3)		(19.7)	(15.6)	(5.5)	
	Contaminated or not tested*	No.	5	9	6		6	4	10	
	(*n* = 20)	(%)	(2.3)	(4.1)	(2.8)		(2.8)	(1.8)	(4.6)	

*16 semen culture contaminated, 4 samples not tested

**Fisher exact test

A strong relationship between culture and CFT positive results (*p* < 0.001) as well as I-ELISA positive results (*p* < 0.01) was also evidenced: almost all rams shedding *B. ovis* in their semen gave I-ELISA positive results (Table 9). The agreement between culture and CFT was almost good (Κ = 0.597; 95 % CI [0.484; 0.709] and good between culture and I-ELISA (45 %) (Κ = 0.710; 95 % CI [0.615; 0.806]).

**Table 9:** Relationship between semen bacteriology and serology

		*B. ovis* culture results				
Serological results		Negative		Positive		
		No.	(%)	No.	(%)	
CFT	Negative	96	(48.7)	25	(12.7)	p < 0.001*
(*n* = 197)	Positive	13	(6.6)	63	(32.0)	Κ = 0.597**
I-ELISA (45 %)	Negative	84	(42.4)	4	(2.0)	*p < 0.001*; Κ = 0.710
(*n* = 198)	Positive	25	(12.6)	85	(42.9)	

*Fisher exact test

**Κ: Cohen’s kappa coefficient

## Discussion

Our study clearly demonstrates that the *B. ovis* infection induces genital lesions and alters the semen quality, leading to an alteration of sexual function of the rams. However this study performed in field conditions on 218 rams included a bias associated to the selection of flocks (voluntary instead of randomly selected) and rams (selected by their owners instead of random), leading to a study sample that could be not representative of the ram population in South-Eastern France.

Another bias is due to the staining kit that probably induced a high percentage of artifactual sperm abnormalities, mainly reflex midpieces, as reported by Kimberling and Parsons [9]. As a consequence, we did not take into account this anomaly for the calculation of the percentage of normal spermatozoa.

The point-score system that we used to classify the rams was adapted from Ley et al. [7]. The clinical and seminal criteria and/or the thresholds selected were close, but slightly different, from those of Ley et al. [7], Kimberling and Parsons [9] and Van Metre et al. [10].

In rams originated from *B. ovis* infected flocks, only 26 % and 37 % of rams had good clinical or seminal score respectively. This result, lower than the 70 % of rams classified as questionable or unsatisfactory breeders in previous studies [7, 10], can be explained by the high prevalence of *B. ovis* infection in our rams population. Furthermore, the low rate of good clinical and seminal scores in the studied population might be due not only to *B. ovis* infection but also to other infectious or non-infectious causes of orchi-epididymitis, as reported previously [2]. A significant relationship was observed between clinical and seminal scores, but this was primarily explained by clinical alterations, not by differences in SC and age. Indeed, SC may be related to genital abnormalities but also to several factors, such as age, breed, season, testicular tone, nutrition, parasite and other concurrent disease status [11].

The comparison of the serological results obtained by the different methods (CFT and I-ELISA) confirms the higher sensibility of the I-ELISA compared to CFT, already reported by Vigliocco et al. [12], Praud et al. [8] and Ridler et al. [13]; moreover, I-ELISA can detect infected rams earlier than CFT [11].

In a recent study, Ridler et al*.* [13] considered that a “suspicious” result is a disadvantage of the *B. ovis* I-ELISA and concluded that rams presenting such a result should be isolated and re-tested 2 to 4 weeks later.

Previously, Praud et al. [8] considered that the use of one or two thresholds can depend on the strategy of control of the disease. In an *a priori* healthy area, a high threshold limits the risk of “false positive” results but, at the opposite, in an infected area, a low threshold increases the diagnostic sensitivity; thus, according to the situations and strategic choices, the class of “doubtful” animals can be grouped with that of “healthy” or “infected”. Under the conditions of our study, I-ELISA-45, with a unique threshold, as proposed by Praud et al. [6], was more sensitive than CFT to detect rams carrying lesions and producing semen of poor quality.

Almost all rams shedding *B. ovis* in their semen were seropositive (mainly in I-ELISA) but approximately 25 % of the seropositive rams were culture-negative for *B. ovis*. This can be due to the intermittent excretion of *B. ovis*, as previously reported [3] and, also, to the fact that the rams were sampled only once [1, 13].

According to previous reports [4, 13, 14], we observed a significant relationship between culture-positive results (*i.e*. excretion of *B. ovis* in semen) and genital alterations or poor semen quality. Although significantly linked, seropositivity and genital tract alterations were not systematically associated, as previously described by Blasco [3], Ridler and West [15] and Van Metre et al. [10]. Moreover, approximately one third of *B. ovis*-shedding rams presented clinical alterations [15], suggesting that *B. ovis* isolation in semen could be more precocious than the detection of epididymitis, as reported by Ridler et al. [13]. Furthermore, clinical alterations may be undetectable in the presence of active excretion of *B. ovis* and serological positive results, especially in chronically infected rams, as described previously by Worthington et al. [16]. In this study, 73 rams showed detectable lesions but 89 gave culture positive results while 81 and 117 gave positive results in CFT and I-ELISA respectively.

## Conclusion

This study performed in *B. ovis* infected flocks of the “PACA” region, confirmed the findings of various previous field or experimental studies. As regards eradication of *B. ovis* infection, the following recommendations could be proposed: in areas with a low-to-medium prevalence, the eradication of CE is possible by using a test-and-slaughter approach, incorporating compounding tests to increase the overall testing sensitivity and reduce the likelihood of false negatives, as proposed by Blasco [3], Ridler and West [15] Praud et al. [8] and Ridler et al. [13]. Such an approach might include a combination of serological (I-ELISA and CFT) and auxiliary tests (genital palpation and, when possible, semen culture). This approach, which must be applied before each breeding season as well as before any ram introduction in free flocks, might allow for more rapid identification and removal of chronic shedders. In areas with a high prevalence*,* this strategy might be economically unsustainable. Despite recently developed *B. ovis* mutant attenuated strains gave promising protecting results in mice [17, 18], the *B. melitensis* Rev.1 vaccine remains up to now the only vaccine with proven efficacy against *B. ovis* infection in rams [2]; therefore, Rev.1 vaccination would be certainly the most economical and practicable tool for midterm control in such situations [3], in particular in areas where export of rams or ram semen is not an economical priority and where the official brucellosis-free status is not a short-term goal.

## Abbreviations

*B. ovis*: *Brucella ovis*
PACA: Provence-Alpes-Côte d'Azur
BCS: Body condition score
SC: Scrotal circumference
CE: Contagious epididymitis
CFT: Complement fixation test
I-ELISA: Indirect enzyme-linked immunosorbent assay

## References

[1] Ficapal A, Jordana J, Blasco JM, Moriyón I (1998). Diagnosis and epidemiology of *Brucella o*v*is* infection in rams. Small Ruminant Res.

[2] OIE (World Organisation for Animal Health), OIE (2014). Ovine epididymitis (*Brucella ovis*). Manual of Diagnostic Tests and Vaccines for Terrestrial Animals.

[3] Blasco JM, Nielsen K, Duncan JR (1990). *Brucella ovis*. Animal Brucellosis.

[4] Carvalho Junior CA, Moustacas VS, Xavier MN, Costa EA, Costa LF, Silva TMA (2012). Andrological, pathologic, morphometric, and ultrasonographic findings in rams experimentally infected with *Brucella ovis*. Small Ruminant Res.

[5] Russell A (1984). Body condition scoring in sheep. In Pract.

[6] Kenyon PR, Maloney SK, Blache D (2014). Review of sheep body condition score in relation to production characteristics. New Zeal J Agr Res.

[7] Ley WB, Sprecher DJ, Thatcher CD, Pelzer KD, Umberger SH (1990). Use of the point-score system for breeding soundness examination in yearling Dorset, Hampshire and Suffolk rams. Theriogenology.

[8] Praud A, Champion JL, Corde Y, Drapeau A, Meyer L, Garin-Bastuji B (2012). Assessment of the diagnosis sensitivity and specificity of an indirect I-ELISA kit for the diagnosis of *Brucella ovis* infection in rams. BMC Vet Res.

[9] Kimberling CV, Parsons GA, Youngquist RS, Threlfall WR (2007). Breeding soundness evaluation and surgical sterilization of the ram. Current therapy in large animal theriogenology.

[10] Van Metre DC, Sangeeta Rao S, Kimberling CV, Morley PS (2012). Factors associated with failure in breeding soundness examination of Western USA rams. Prev Vet Med.

[11] Ridler AL, Smith SL, West DM (2012). Ram and buck management. Anim Reprod Sci.

[12] Vigliocco AM, Silva Paulo PS, Mestre J, Briones GC, Draghi G, Tossi M (1997). Development and validation of an indirect enzyme immunoassay for detection of ovine antibody to *Brucella ovis*. Vet Microbiol.

[13] Ridler AL, Smith SL, West DM (2014). Seroconversion and semen shedding in rams experimentally infected with *Brucella ovis*. New Zeal Vet J.

[14] Kott RW, Halver GC, Firehammer B, Thomas VM (1988). Relationships between *Brucella ovis* semen culture and various semen and serology parameters. Theriogenology.

[15] Ridler AL, West DM (2011). Control of *Brucella ovis* Infection in Sheep. Vet Clin N Am-Food A.

[16] Worthington R, Stevenson B, De Lisle G (1985). Serology and semen culture for the diagnosis of *Brucella ovis* infection in chronically infected rams. New Zeal Vet J.

[17] Sancho P, Tejedor C, Sidhu-Muňoz RS, Fernández-Lago L, Vizcaíno N (2014). Evaluation in mice of *Brucella ovis* attenuated mutants for use as live vaccines against *B. ovis* infection. Vet Res.

[18] Soler-Lloréns P, Gil-Ramírez Y, Zabalza-Baranguá A, Iriarte M, Conde-Álvarez R, Zúñiga-Ripa A (2014). Mutants in the lipopolysaccharide of *Brucella ovis* are attenuated and protect against *B ovis* infection in mice. Vet Res.

